# Controversy on the toxic nature of deep eutectic solvents and their potential contribution to environmental pollution

**DOI:** 10.1016/j.heliyon.2022.e12567

**Published:** 2022-12-24

**Authors:** Guillermo Martínez Martínez, Gabriela Guillena Townley, Rosa María Martínez-Espinosa

**Affiliations:** a Biochemistry and Molecular Biology Division, Agrochemistry and Biochemistry Department, Faculty of Sciences, Ap. 99, E-03080, Alicante, Spain; b Multidisciplinary Institute for Environmental Studies (IMEM), University of Alicante, Ap. 99, E-03080, Alicante, Spain; c Organic Chemistry Department and Organic Synthesis Institute (ISO), University of Alicante, Ap. 99, E-03080, Alicante, Spain

**Keywords:** Deep eutectic solvents, Natural deep eutectic solvents, Ecotoxicity, Biodegradability, Sustainability

## Abstract

Deep eutectic solvents (DES) are promising reaction media where interesting catalytic processes can be carried out. In theory, most of these mixtures are environmentally friendly, being an alternative to traditionally pollutant organic solvents used in several processes related to organic chemistry and biotechnology. However, recent studies show contradictory results regarding their toxicity. The method selected to perform toxicity studies could be significantly conditioned by some of the physical properties displayed by the DESs. Also, the metabolic capabilities of the organisms/cells used to monitor their toxicity are influenced by their physical properties. In this review, relevant physical-chemical properties for toxicity studies are summarized. The advantages/disadvantages of the used tests to monitor their toxicity and biodegradability in connection with the chosen organisms/cells are discussed, shedding light on their limitations. These findings could be taken as a starting point for designing more accurate DESs toxicity studies covering a wider spectrum of organisms and cells to be used as biomodels to monitor environmental pollution caused by DESs.

## Graphical abstract

**Figure:**
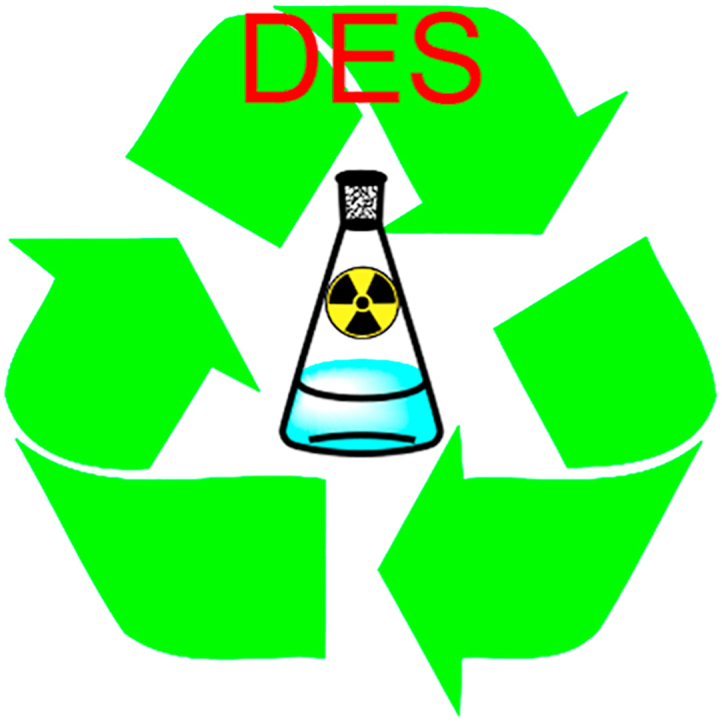

## 1 Introduction

The so-called green technologies are englobed in a wider and interdisciplinary research area known as Green Chemistry, which pursues to produce chemicals in a clean, safe, efficient, and sustainable manner [1]. With this aim, the reduction and/or elimination of the use or generation of chemicals, which are toxic and/or harmful to the environment, has to be considered when designing, developing, or implementing any chemical processes.

In this context, the implementation of catalytic processes and the choice of the appropriate solvent to be used as reaction media are crucial. Around 80% of the total volume of chemical components involved in a process corresponds to the solvent [2]. Most of them are volatile organic compounds (VOC) connected to health challenges (including human and ecological toxicity issues) and limitations regarding waste management, due to their inherent toxicity and high volatility [3]. Therefore, the development of cost-effective and environmentally friendly solvents has become an urgent objective of analysis in the chemical industry. Replacement of VOCs avoids the emission of toxic or flammable vapours, increasing the efficiency of the process from an economic and environmental point of view [4]. Some alternatives to VOCs, such as water [5], ionic liquids (ILs) [6, 7], glycerol and derivatives [8] have been used in chemical processes. However, the use of these types of solvents is still limited by many environmental and toxic problems, such as their high pollution index and/or low biodegradability [9].

To overcome these limitations and improve the applicability, Deep Eutectic Solvents (DESs) [10, 11] were introduced in 2001 [12]. Unlike ILs, DESs are mixtures and not pure compounds. Studies about their physicochemical properties [13] pointed out that they are a new type of liquid fluids with interesting applications in several research fields [14], such as analytical chemistry [15, 16], electrochemistry [17, 18], polymer science [19], biotechnology [20, 21], organic synthesis [22, 23], material sciences [24] and biomass processing [25].

**Figure 1:**
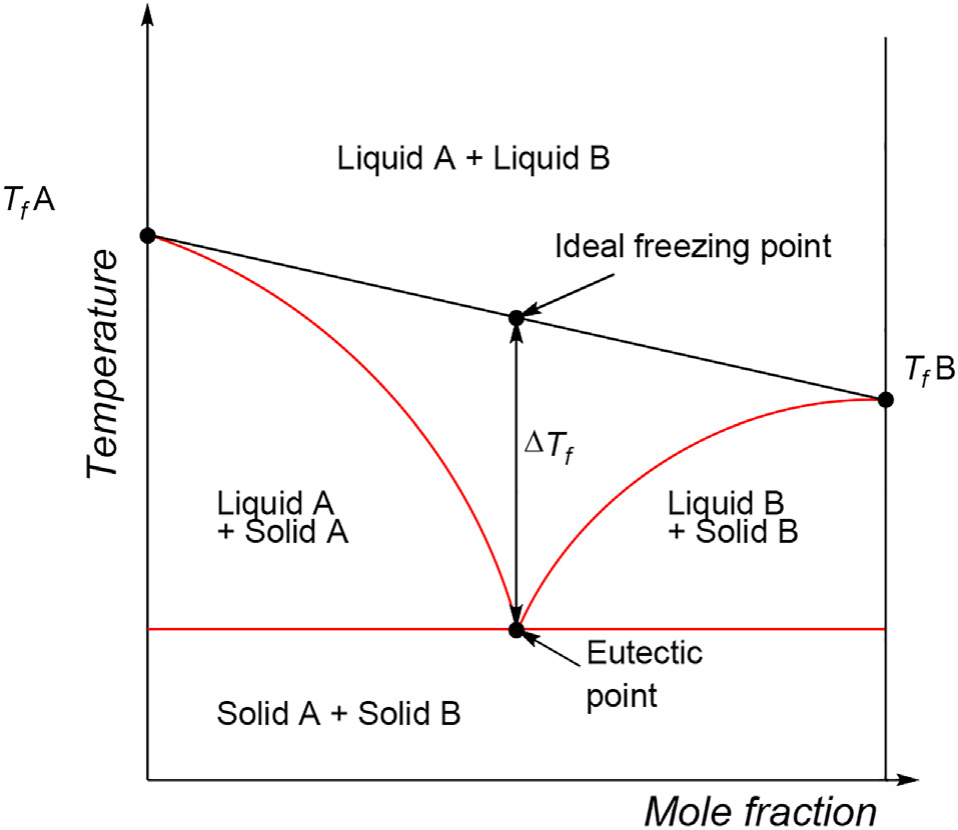
Schematic representation of a DES phase diagram.

A DES is formed by the combination of two or more compounds, one acting as a hydrogen bond donor (HBD) with the other behaving as an acceptor (HBA), that form a liquid eutectic mixture, at a certain molecular ratio with a melting point lower than that of the ideal mixture [26]. The temperature depression (${\Delta T}_{f}={\Delta T}_{f\left(real\right)}-{\Delta T}_{f\left(ideal\right)}$) (Figure 1) should be defined as the difference between the measured freezing point of a mixture at the eutectic composition ${[\Delta T}_{f\left(real\right)}$)] and the theoretically predicted freezing point for an ideal mixture [(${\Delta T}_{f\left(ideal\right)}$)] [27]. Generally, it is assumed that charge delocalization due to hydrogen bond type interactions [28] is the key intermolecular force behind the melting point depression, together with steric effects and ionic contributions from the anion/cation pair. Thus, the stronger the hydrogen bond is, the deeper the decrease in melting point occurs. This increase in strength is counterbalanced by a reduction of other interactions [29]. For instance, ChCl:urea (1:2) shows a $T_{f}$ of 12 °C [30]. This temperature is very far from each of the melting points of Choline chloride (ChCl) and urea (U), 302 °C and 133 °C, respectively.

Compared to ILs, the advantages of DESs are their lower cost of production, simpler preparation without further purification; higher biodegradability and less toxicity [31]; and high tunability due to the myriad of possible binary combinations of HBDs/HBAs. Additionally, DESs display high thermal stabilities, low volatility, and low vapour pressures. For all these reasons are considered “designer” green solvents [32].

Due to all the possible combinations, DESs have been classified into four different types: Type I (Metallic chloride + quaternary ammonium salt, e.g. ChCl:ZnCl_2_), Type II (Metal chloride hydrate + ammonium salt, e.g. ChCl:CrCl_3·_6H_2_), Type III (HBD + quaternary ammonium salt, ChCl:urea) and Type IV (Metal chloride hydrate + Hydrogen bond donor e.g. ZnCl_2_:urea) [33]. However, there are some described DESs that do not fit in this classification. Formed by the mixture of non-ionic, molecular HBAs and HBDs species (Type V), most of these mixtures behave ideally and therefore are not true DESs [34].

**Chart 1:**
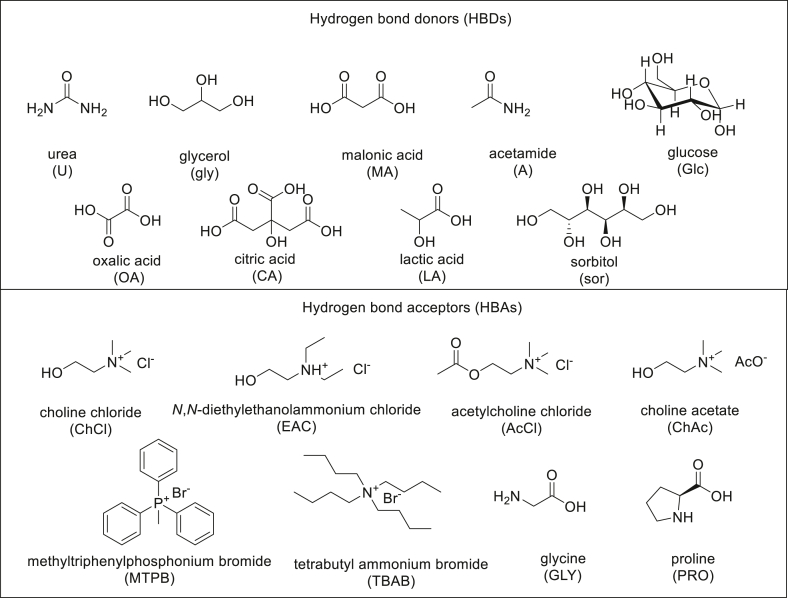
Main hydrogen bond donors (HBDs) and acceptors (HBAs) used in the synthesis of DESs.

Particularly versatile are DESs of type III due to the wide range of HBD and HBA available, their reported applications being larger than for other types of DESs (Chart 1).

The broad number of described DESs, especially those belonging to types III and V, has led to the emergence of some subfamilies according to their nature, physical properties, or applications. Thus, NADESs [35] were introduced as a new generation of DESs, based on their hypothetical biological function in Nature [36, 37]. When one of the components of the DES mixtures is a bioactive or a pharmaceutical ingredient, these have been defined as a therapeutic deep eutectic system (THEDES) [38]. Similarly, if one of the components is an acid or an amino acid, the terms acidic deep eutectic solvent (ADES) [39] or amino acid-based DES (AADES) [40] are coined.

Most of the types and subfamilies of DES presented so far, are hydrophilic [41]. However, to overcome limitations related to their application in aqueous media, water-immiscible DES, known as hydrophobic deep eutectic solvents (HDESs) have been recently introduced. Studies related to these HDESs have expanded the possibilities and applications of this type of solvent [42].

Due to their wide range of applications in diverse areas, DESs have attracted attention at the industrial and academic levels. This has resulted in an increasing number of studies published mainly in the last two decades.

In all these applications, the role of DESs as non-toxic and biodegradable solvents has been highlighted. However, there is a lack of toxicological and environmental studies confirming these facts. This assumption was wrongly based on the benign properties of each of the individual components, particularly when NADES are applied [43]. However, the possible synergistic effect between the components of DESs, the impact on their hazardous properties and the ecological footprint of these eutectic mixtures should not be neglected.

The aim of this review, complementary to others that have recently appeared in the literature [32], is to review whether DESs are non-toxic and environmentally friendly, focusing on the methods and approaches employed to conduct these studies. These results and the selected method could be severely compromised by some of the physical properties of the DESs. The results would be presented based on the chosen organism to conduct the toxicity assays, the nature of the tests performed, and the relation between the achieved results and the expected application of the DESs.

## 2 Physical properties of Dess relevant for environmental toxicity and biodegradability studies

**Figure 2:**
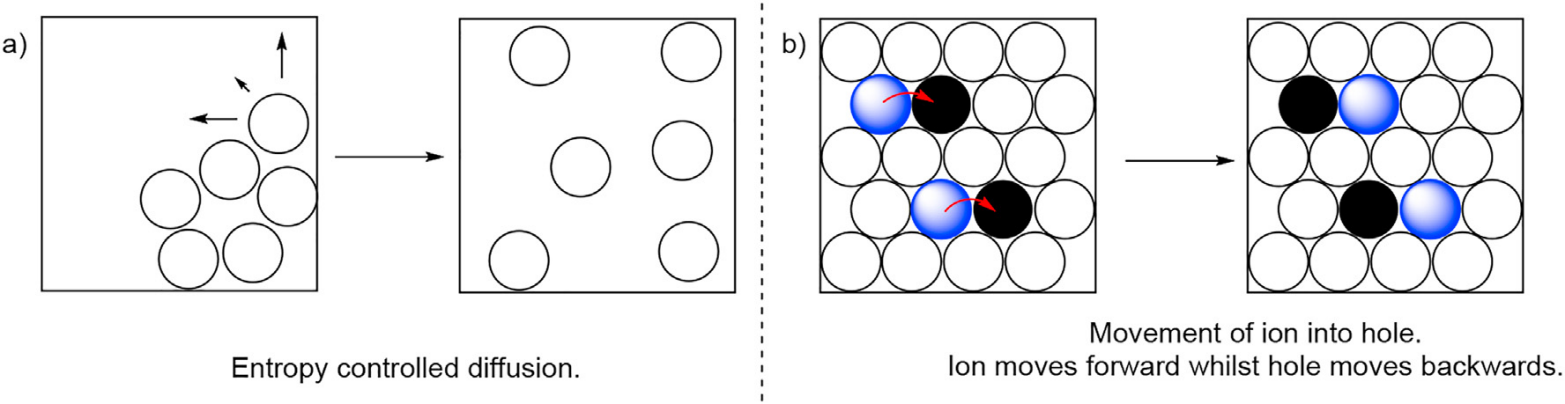
Difference in the molecular movement in a) a molecular liquid and b) DES.

DES's physical properties are dependent on the type of interactions established between its components. Also, the composition of DESs determines their specific applications. For instance, HDESs are mostly applied to food extraction and pharmaceutical and medicinal purposes. This type of application needs a careful study of its ecotoxicity and biodegradability. However, studies related to these aspects are scarce and generally, if performed, have not taken into account their physical properties, giving misleading results about their toxic and biodegradable nature. The main physical properties relevant to those studies are viscosity, density, water content and pH. However, more recently it has also been described that reported values of physiochemical properties differ between studies involving the same DESs due to their thermal instability. Thus, it was observed that long-term thermal stability was mainly related to the thermal stability of DES starting materials [43]. The viscosity of any liquid is one of the most important properties that must be considered related to its application. Eutectic mixtures with low viscosity values are preferable from an environmental standpoint. But DESs, like ILs, are much more viscous solvents than VOCs. Except for ChCl:EG mixtures, most DESs have a high degree of viscosity (greater than 100 cP) at room temperature [44]. The viscosity of DESs is determined by the chemical nature of the HBA/HBD components (nature; molar ratio, etc.), the temperature and the water content. For example, ChCl mixtures derived from sugars, such as sorbitol [45], or carboxylic acid, such as malonic acid (MA) [46] as HBD, produce an increase in the viscosity of the mixtures. This is attributed to the presence of an extensive network of hydrogen bonds between the two components. This causes lower mobility of the species within the DES, influencing their molecular transport. Whilst molecular liquids are diffusion-controlled, allowing molecules to move into vast amounts of empty space, components in DESs and ILs are more closely held together in a hydrogen-bonded network containing empty spaces or holes (Figures 2a and 2b) [47].

Although this apparent complexity, a general viscosity model to estimate their viscosities, based on the free volume theory coupled with the associated equation of state [48], or a simple predictive viscosity model for DESs based on a database covering 156 deep eutectic solvents of different nature, has been proposed [49].

DESs have a higher density than water, varying between 1.0 and 1.35 g/cm^3^ at room temperature. The significant differences in the densities of various DESs area due mainly to their molecular organization. Therefore, different densities are found when the constituent molecules are changed. For example, glycerol-based DES have a higher density compared to ethylene glycol(EG)-based DES and benzyl ammonium chloride salts as HBA [50]. The presence of an increasing number of hydroxy groups leads to more hydrogen-bonding formation, which decreases the free volume available. Also, the molar ratio between the organic salt and the HBD has an obvious effect on the density. The addition of ChCl to glycerol (gly) led to a significant decrease in the density of the ChCl:gly mixtures, which can be explained in terms of free volume [51].

Another significant factor that influences DESs density is temperature. These densities can be accurately estimated as a function of temperature by using a simple relation based on genetic programming (GP) and a large data bank of experimental data from 149 DESs of different natures [52].

Most DESs contain coordinated hydrogen-bonded cations and anions, which are strongly water-miscible and hygroscopic. This inherent absorption of water [53] would affect their physicochemical properties, such as the melting point, density, viscosity, refractive index, speed of sound and pH [54]. Thus, adding small amounts of water, the DES structure is still preserved and an exponential decrease in its viscosity is observed [55]. This effect on the DES nanostructures has been carefully studied by adding water to ChCl:U mixtures, showing that up to 42 wt% H_2_O can be added without affecting its nanostructure. Probably, this is due to the solvophobic sequestration of water molecules into nanostructured domains around cholinium cations. This becomes unfavourable at higher water content, leading to a disruption of the DES structure [56].

The negative value of the excess molar volume (*V*_m_^E^) observed for several aqueous ChCl-based DESs resulted from a synergistic interaction between the DES and water along with the interstitial accommodation of small water molecules within the DES. The presence of strong intermolecular hydrogen bonding between the Cl–H_2_O, OH–H_2_O of ChCl, and HBD-H_2_O in the water-rich region contributes to the highest deviation from the optimum in the *V*_m_^E^. Meanwhile, in the DES-rich region, intramolecular hydrogen bonding among the DES-DES and water-water molecules is dominant, water molecules not being enough to sufficiently hydrate the entire DES molecules [57, 58].

The pH is one of the most critical properties to consider when DESs are designed for specific applications. Also, the intrinsic pH value of a given DES is crucial when its toxicity is evaluated using living organisms such as bacteria. As DES are formed by mixing compounds with Lewis or Brønsted acidic and basic nature, their pH depends on the relative acidity of their anionic and cationic constituents [59]. A large difference between the pH values of the two most used DES such as ChCl:U and ChCl:EG (1:2) mixtures have been encountered at 20 °C, 10.387 and 4.676, respectively. These values indicate that ChCl:U has a basic character with a relatively high concentration of hydroxy ions, while ChCl:EG has an acidic character, probably due to more extensive hydrogen bond [60].Although is generally accepted that the difference in the observed pH is mainly influenced by the acidic character of the HBD [61], also the HBA has an effect on these values as it has been shown for triethylene glycol (TEG)-based DESs [62]. The temperature influences the pH value. For instance, when the pH of several ChCl:D-glucose mixtures was measured at 25–45 °C, the pH was almost neutral. However, raising the temperature to 85 °C, led to a pH decrease showing a slight linear dependence with increasing temperature [63].

Recent studies have highlighted the role of DESs as non-toxic and biodegradable compounds based on the benign properties of each component [13], especially for those derived from choline salts or other natural compounds [64, 65]. However, almost all these studies do not have into consideration the possible synergistic effect between its components, which will undoubtedly have a considerable impact on the properties of these eutectic mixtures.

## 3 Biodegradability of DESs

A close bottled test is the most common method to determine the potential for biodegradation of chemical compounds. In this type of test, DES is added to aqueous solutions in which microorganisms are also inoculated and oxygen depletion is measured. To be considered valid, 60% of the theoretical oxygen demand must be reached within the first 14 days of the experiment. Furthermore, a toxic compound is easily degradable if the 60% level is reached within the first 28 days [66].

A study on the biodegradability of 8 different DESs using the closed bottle test showed that only 2 could be considered truly biodegradable (Table 1, entry 1) [31]. At a comparative level, those DESs based on ChCl displayed a greater degradation rate than those based on ChAc, their biodegradability rate being similar to conventional ILs. All the tested DESs showed a significant toxic effect on selected living organisms (short life of H. Sinensis and the reduced length of the roots of *A. Savitum* in comparison with controls), observing deformities in their structure. ChCl-based DESs had toxic effects bigger than those based on ChAc, following the toxicity trend of ChCl and ChAc salts (Table 1, entry 1). In another study, three DESs based on ChCl presented a percentage of oxygen demand greater than 60% (Table 1, entry 2). These results could be attributed to the biodegradable nature of the individual components [67].

**Table 1:** Degradability results of studied DESs using the closed bottle test.

Entry	DES components	%Degradability (14 d)	Ref
1	ChCl:U	> 60	[31]
	ChCl:A	> 60	
	ChCl:gly	> 40	
	ChCl:EG	> 20	
	ChAc:U	> 40	
	ChAc:A	> 40	
	ChAc:gly	> 30	
	ChAc:EG	> 30	
2	ChCl:gly	> 90	[67]
	ChCl:Glc	> 70	
	ChCl:OA	> 60	
3	ChCl:gly	> 90	[68]
	ChCl:U	> 80	
	ChCl:EG	> 70	
	EAC:gly	> 70	
	EAC:EG	> 70	
	EAC:ZnCl_2_	> 60	
	EAC:MA	> 60	

HBAs also play an important role in the degradability of DESs. Thus, ChCl-based DESs were compared with those based on *N,N*-diethylethanolammonium chloride (EAC). All of them were defined as truly biodegradable (Table 1, entry 3), but those based in EAC were less degradable. This fact was attributed to the presence of the methyl groups in ChCl that favour their biodegradation compared to the ethyl groups in EAC. In addition, the presence of more hydroxyl groups in the HBD counterpart notably improved the biodegradability of the DES [68].

However, there are not yet enough studies to draw an evident conclusion about the behaviour of DESs in terms of biodegradability. Therefore, more research and development are needed in this area.

## 4 Tests used to analyse Dess toxicity: advantages and disadvantages

The nature of the test used to analyze the toxicity of a compound is crucial to obtain accurate and reproducible results. Studies about DESs toxicities are mainly based on cytotoxicity or cell viability tests (using human cell lines) (Table 2) and toxicity or antimicrobial activity tests (Table 3).

**Table 2:** Cytotoxicity assays of studied DESs.

Entry	DES components	Assay	Cell lines	Results	Ref
1	ChCl:MA ChCl:CA ChCl:LA ChCl:Fru ChCl:Xyl ChCl:Man	MTT	CCO	ChCl-based DESs are less toxic than ILs.	[71]
2	ChCl:gly ChCl:Glc ChCl:OA	MTT	CCO, MCF-7	ChCl: gly and ChCl:Glc EC_50_ > 10mM ChCl:OA EC_50_ 1.64 ± 0.14mM for CCO and 4.19 ± 0.19 mM for MCF-7	[67]
3	ChCl:gly ChCl:U ChCl:G ChCl:TEG	MTT	MCF-7, A375, OKF6, HT29, H413, PC3, HepG2	Increased values of IC_50_ were obtained for ChCl:TEG, ChCl:gly, ChCl:EG, ChCl:U with OKF6, MCF-7, A375, HT29 and H413, respectively	[72]
4	ChCl:Glc ChCl:Frc ChCl:Suc ChCl:gly ChCl:MA	MTT	MCF-7, HelaS3, CaOV3, B16F10	Increased values of IC_50_ were obtained for MCF-7, HelaS3, B16F10 with ChCl:MA, ChCl:Suc, ChCl:Frc, ChCl:Glc, ChCl:gly respectively	[75]
5	ChCl:Glc ChCl:Frc EAC:TEG	MTT	HelaS3, PC3, A375, AGS, MCF-7, WRL-68	98 ≤ EC_50_ ≥ 516 mM for ChCl: Glc and ChCl: Frc, 34 ≤ EC_50_ ≥ 120 mM for EAC: TEG EC_50_ values ChCl:Glc and ChCl:Frc >> aqueous solutions of ChCl, Frc and Glc	[76]
6	28-based ChCl DESs combined with alcohols, sugars, and derivatives	MTT and QSAR analysis	HEK-293	IC_50_ values in the range of 3.52–75.46 mM	[77]
7	ChCl:AsC	MTS	L929	EC_50_ 1.47 ± 0.48 mM Above EC_50_, the cells presented an apoptotic morphology	[79]
8	Menthol:SA Menthol:MyA Menthol:LaA	MTS	HaCaT	EC_50_ 5.569 ± 0.326 mM for menthol:LaA < Menthol:MyA˂ Menthol:SA	[80]
9	LIM:CpA LIM:Menthol LIM:IBU (1:4) and (1:8)	MTS	Caco-2	EC_50_ 10.50 ± 0.883 mM for LIM:IBU (1:4)> LIM:IBU (1:8)˃LIM:menthol˃LIM:CpA LIM:IBU showed potential as a drug delivery system in anti-cancer therapies	[81]
10	CA:Ethambutol:H_2_O (2:1:10) CA:ARG:H_2_O (1:1.7) to (2:1:9)	MTS	Caco-2	IC_50_ 0.1085 for CA:ARG:H_2_O (1:1:7) comparable to ARG	[83]

**Table 3:** Toxicity results using Microtox and related methodologies for some studied DESs.

Entry	DES components	Organisms used for toxicity assay	Method	Conclusions	Ref
1	ChCl:AA, ChCl:CA, ChCl:LA, ChCl:GA	*Allivibrio fischeri.*	Microtox	DES toxicity > components. Toxicity determined HBD concentration. ChCl:AA < ChCl:LA < ChCl:GA < ChCl:CA	[85]
2	ChCl:gly, ChCl:EG ChCl:PA ChCl:U ChCl:1-propanol ChCl:1,2-propanediol	*Allivibrio fischeri.*	Microtox	ChCl:U and ChCl:1-propanol EC_50_ > components. All DESs showed an antagonistic effect with the exception ChCl:PA.	[86]
3	[N_1111_]Cl:EG, [N_1111_]Cl:1-propanol, [N_2222_]Cl:EG, [N_2222_]Cl:1-propanol, [N_3333_]Cl:EG, [N_3333_]Cl:1-propanol,	*Allivibrio fischeri.*	Microtox	Toxicity values [N_1111_]Cl-DES < [N_2222_]Cl-DES < [N_3333_]Cl-DES. Synergism for[N_3333_]Cl-DES and [N_2222_]Cl:EG	[88]
4	ChCl:gly ChCl:EG ChCl:U ChCl:gly:H_2_O ChCl:EG:H_2_O ChCl:U:H_2_O	*Allivibrio fischeri* *Selenastrum capricornatum* *Daphnia magna*	Microtox, OECD test201 [91] OECD test202 [92]	ChCl:U˃ ChCl:gly˃ ChCl:U:H_2_O˃ ChCl:EG˃ ChCl:EG:H_2_O˃ ChCl:gly:H_2_O for bacteria, ChCl:U:H_2_O˃ ChCl:EG:H_2_O˃ ChCl:gly:H_2_O˃ ChCl:gly˃ ChCl:U˃ ChCl:EG for algae ChCl:U˃ ChCl:U:H_2_O˃ ChCl:gly:H_2_O˃ ChCl:EG˃ ChCl:EG:H_2_O˃ ChCl:gly for crustaeans	[90]

The methodology called MTT (standing for 3-(4,5-dimethyl-2-thiazolyl)-2,5-diphenyl-2H-tetrazolium bromide) is a colourimetric test evaluating the cellular metabolic activity [69, 70]. This assay determines mitochondrial activity, which is correlated with the number of viable cells, allowing to measure in vitro cytotoxic effects in cell lines. When DESs based on ChCl linked to sugars, alcohols and organic acids were studied in channel catfish ovary (CCO) fish cell line, low cytotoxicity was found compared to ILs (Table 2, entry 1) [71]. In a more detailed study, the cytotoxic profile of three DESs was studied on fish cell line (CCO) and the human breast adenocarcinoma cell line (MCF-7), showing that ChCl:OA presented significantly higher toxicity (EC_50_ < 5 mM) compared to the others (EC_50_ > 10 mM) (Table 2, entry 2) [67]. The cytotoxicity of ChCl mixtures was investigated using different cell lines, showing that the cytotoxicity effect varied with the cell lines (Table 2, entry 3) [72]. To achieve the optimal therapeutic effect and to verify that non-target cells are less affected, the DESs cytotoxicity was compared with OKF6 as a non-target cell line. Results showed no DNA fragmentation, but DES-treated MCF-7 cells increased the lactate dehydrogenase (LDH) release and triggered the reactive oxygen species (ROS) production [73], evidencing that the cell death was caused through another mechanism, rather than apoptotic DNA fragmentation.

In another study, different ChCl-based NADESs were evaluated by MTT assay together with a conductor-like screening model for real solvent (COSMO-RS) [74] software for the analysis of the cytotoxic mechanism [75]. The results showed the same trend of cytotoxicity for all cell lines tested, with the ChCl:MA mixture being the most toxic one (Table 2, entry 4). A correlation between the cytotoxicity and viscosity values of the DESs was observed, with the most viscous being the most toxic. Modelling through COSMO-RS the interactions between the studied NADESs and some phospholipids found in cellular membranes pointed out that NADESs interacted strongly with the cell surfaces, suggesting a hypothetical cytotoxic mostly based on cellular aggregation.

NADESs (ChCl:Frc and ChCl:Glc) were less toxic than EAC:TEG DES for different cell lines (Table 2, entry 5) [76]. However, the NADESs EC_50_ values were significantly higher than those of the aqueous solutions of their individual components. An assessment of membrane permeability and redox stress showed that even though NADESs are more permeable by increasing membrane porosity, the DES stimulated ROS synthesis to a greater extent than NADES.

The cytotoxicity results obtained against human embryonic kidney HEK-293 cell line using the MTT methodology applied to 28 ChCl-derived DESs and different alcohols, sugars and their derivatives as HBD were used to evaluate the effect of structural parameters on the cytotoxicity by using quantitative structure-activity relationship (QSAR) analysis (Table 2, entry 6) [77]. The studied mixtures displayed more cytotoxicity than their corresponding starting materials but were found to be less toxic than the corresponding ILs. The cytotoxicity increased by increasing the rotatable bond number (RBN), related to the flexibility of molecules and their capability to penetrate through the cell membrane and the mean atomic van der Waals volume (Mv) of HBD compounds. These results demonstrated that constitutional descriptors and the DESs HBA: HBD molar ratio have an important role in cytotoxicity.

To determine the cytotoxicity of some THEDESs, the MTS assay was used. In this assay, the conversion of 3-(4,5-dimethylthiazol-2-yl)-5-(3-carboxymethoxyphenyl)-2-(4-sulfophenyl)-2H-tetrazolium bromide to purple formazan in the presence of phenazine methosulfate is measured allowing to evaluate the metabolic activity and viability of cells [78]. This assay was applied to study the cytotoxicity against murine fibroblast cell line L929 of ChCl:ascorbic acid (AsC) founding intermediate EC_50_ values (Table 2, entry 7) compared to their components [ChCl (lower cytotoxic) and AsC (higher cytotoxicity)], pointing out a synergistic effect between the individual compounds [79]. The cell viability varied in a dose-dependent manner decreasing at higher concentrations of THEDES, probably due to a higher amount of AsC causing a gradual drop in pH and an increased value of viscosity.

The MTS assay against human epidermal keratinocyte HaCaT cell line of several menthol and saturated fatty acids [e.g., stearic acid (SA), myristic acid (MyA), and lauric acid (LaA)] based THEDESs, showed that menthol:LaA displayed the highest cytotoxicity, similar to that of pure menthol [80]. Meanwhile menthol:MyA and menthol:SA had lower cytotoxicity than the individual components (Table 2, entry 8).

THEDESs formed by combination based on limonene (LIM):capric acid (CpA) (1:1), LIM:menthol (1:1), Ibuprofen (IBU) [(1:4) and (1:8)] were used to assess cell cytotoxicity against the human colon epithelial cancer Caco-2 cell line and antiproliferative effects. Results showed that LIM:IBU (1:4) was the less cytotoxic mixture (Table 2, entry 9) [81]. All studied THEDESs demonstrated antiproliferative properties with HT29, but only LIM:IBU (1:4) inhibited HT29 proliferation without comprising cell viability. Therefore, this system was chosen to further assess anticancer properties by analyzing the effects on the cell cycle, apoptosis, intracellular ROS and NO production. The lowest tested concentration of LIM:IBU (1:4) protected HT29 cells from oxidative stress, demonstrating anti-inflammatory effects by inhibiting ROS and NO production. Results of these anticancer properties suggested that apoptosis should be induced via caspase-2 or caspase-9 pathways [82].

Based on the MTS assay used to study the cell viability of tertiary DES mixtures against Caco-2 cells [83], the cytotoxicity was affected by the presence of citric acid in a dose-response manner (Table 2, entry 10). This fact probably was due to the highly acidic nature of the studied DESs that increased with the amount of citric acid, and it was concordant with the obtained IC_50_ values of the individual components.

Regarding toxicity tests, the so-called Microtox test is also frequently used [84]. This test is based on a photometric technique using the inhibition of the luminescence of the marine bacterium *Allivibrio fischeri*, in the presence of toxic compounds. This bacterium is overly sensitive to a wide variety of toxic compounds, even when they are present at low concentrations. The incubation of this bacterium is carried out with diluted samples of the different DESs, evaluating the inhibition of luminescence compared to a control sample. This type of test has been used to evaluate the toxicity of the DESs prepared by a combination of ChCl with compounds like acetic (AA), citric (CA), lactic (LA) and glycolic (GA) acids with different molar ratios (2:1, 1:1 and 1:2) (Table 3, entry 1) [85], or ethylene glycol, glycerol, 1,2-propanediol, propionic acid (PA), 1-propanol, and urea in different molar ratios (Table 3, entry 2) [86]. As Microtox tests are carried out in diluted aqueous conditions, the principles of mixtures toxicity theory were applied to consider the relative toxicity of the mixture components. The use of dimensionless toxic units (TU) [87] to express the toxic strength of the DESs was pointed out. The mixture can be classified into additivity when the toxicity of the mixture is equal to the sum of the toxicities of the individual components; synergistic if the toxicity of the mixture is greater than the sum of the individual toxicities; or antagonistic when the toxicity of the mixture is less than the addition of that of the components. For all studied DESs, a clear antagonist effect was encountered independently of the molar ratios, except for ChCl:PA, where a synergic interaction was found for molar ratios 2:1 and 4:1 (Table 3, entry 2). These results pointed out that DESs are less toxic than either of their starting materials dosed separately.

Applying the Microtox test to tetramethylammonium chloride ([N_1111_]Cl), tetraethylammonium chloride ([N_2222_]Cl) and tetrapropylammonium chloride ([N_3333_]Cl) as HBAs, combined with glycerol and 1-propanol as HBD, higher toxicity of [N_3333_]Cl-based DES was found compared to that of [N_1111_]Cl- and [N^2222^]Cl-based DES (Table 3, entry 3) [88]. For these DESs, the concentration addition (CcA) and independent action (IA) models can be formulated and applied [89]. When the toxicity data of the DESs were fitted to CcA and IA models with deviations describing synergism/antagonism, dose-ratio and dose-level effects, a better adjustment for the experimental data from five out of the six DESs investigated was found with the IA model. This type of toxicity test has been used in bacteria (*A. fischeri*), crustaceans (*Daphnia magna*), and algae (*Selenastrum capricornatum*) as biomodels to test three pure DESs (ChCl:gly, ChCl:EG and ChCl:U) and their tertiary mixtures with water (Table 3, entry 4).^900^ Results showed that none of the mixtures was toxic for the environment, ChCl:U being the most toxic DES for bacteria and crustaceans, whereas ChCl:gly was the most toxic for algae.

**Figure 3:**
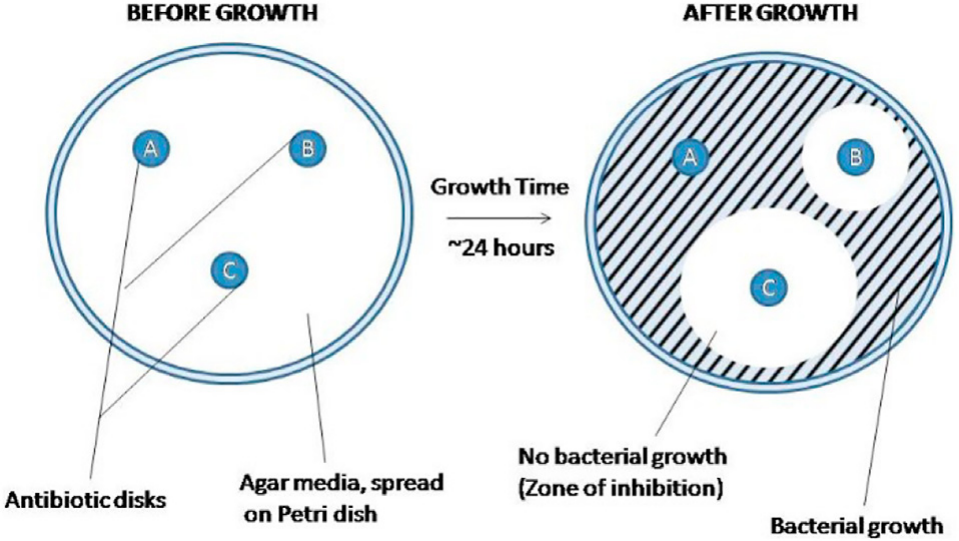
Schematic representation of an antibiogram [96].

The main methodology used to test DES's toxicity was the antibiogram or plaque diffusion method (Figure 3) [93, 94]. These studies are generally carried out using different strains of Gram-positive and Gram-negative due to differences in terms of membrane structure [95]. However, the use of this type of methodology is not without a drawback: DESs are compounds with a high viscosity, which could restrict their diffusion, impairing the reliability of results.

As an example, filter paper diffusion assay [97] was used to assess the toxicity of methyltriphenylphosphonium bromide (MTPB)-based DES [98] using two Gram-positive bacteria and two Gram-negative bacteria. The toxicity observed with the DESs was higher than each of the components. These observations were attributed to the hydrogen bonding between the HBD and the anion of the salt, which not only affected the physical properties, but also the chemical structure of the mixture. Therefore, it can be concluded that there is a synergistic effect between the two components of the DES mixture, resulting in an increase in toxicity in the species analysed (Table 3, entry 1). The disk diffusion method was used to evaluate the toxicity of ten NADES based on ChCl, Betaine (B) and CA against Gram-negative, Gram-positive bacteria and yeast (Table 3, entry 2) [99]. Citric acid (CA) containing NADES showed the biggest growth inhibition, probably due to the high acidity. Furthermore, the strongest inhibitory effect cytotoxicity effect against HeLa, MCF-7 and HEK293 was also found with ChCl:OA most likely due to the formation of calcium oxalate crystals inside the cells. The antioxidative activity of the tested NADES was determined by the oxygen radical absorbance capacity (ORAC) method [100], which relays on assessing the effect of presumed antioxidants by measuring fluorescence quenching. The tertiary DES B:MA:PRO showed the highest ORAC value together with a beneficial effect on the proliferation of tested cell lines. In another study, the disk diffusion method was applied to determine the antibacterial activity of capric acid (CpA) based DES combined with saturated fatty acids with different chain size lengths: lauric acid (LaA), myristic acid MyA), stearic acid (SA) (Table 4, entry 3) [101]. However, the determination of the minimal inhibitory concentration (MIC), minimal fungicidal concentration (MFC) and minimal bactericidal concentration (MBC) in suspension culture, was hampered due to the turbidity of lipidic solutions and insolubility in water. The achieved MIC/MBC/MFC results demonstrated that the inhibition halos in the disk assay were most likely influenced by the diffusion ability of the compounds and incubation temperature. The antimicrobial activity followed the expected trend CpA:LA˃CpA:MA ˃CpA:SA, pointing out that medium and long-chain fatty acids generally displayed a higher antibacterial activity towards Gram-positive bacteria.

**Table 4:** DESs toxicity results using diffusion methods.

Entry	DES components	Organisms used for toxicity assay	Main results	Ref
1	MTPB:gly MTPB:EG MTPB:TEG	*Gram(-): Escherichia coli* and *Pseudomonas aeruginosa*. *Gram(+): Artemia salina*, *Bacillus subtilis*, *Stapylococcus aureus*,	DES toxicity > components. DES showed anti-bacterial activity	[98]
2	ChCl with U, OA, Xyl and Sor B with Glc, MA: PRO, MA:Glc, CA with PRO, Glc:gly,	*Gram(-): Escherichia coli, Proteus mirabilis, Salmonella typhimurium and Pseudomonas aeruginosa* *Gram(+): Staphylococcus aureus* *Candida albicans*	No inhibition of bacteria with ChCl:Xyl, ChCl:Sor and B:Glc ChCl:U was effective only against *E. coli* *C. albicans* was only inhibited by ChCl:OA	[99]
3	CpA:LaA CpA:MyA CpA:SA	*Gram(-): Pseudomonas aeruginosa* and *Escherichia coli* *Gram(+): Staphylococcus aureus*, *Staphylococcus epidermis,* *Candida albicans*	No inhibition against Gram-negative Misleading results for Gram-positive bacteria and yeast due to the diffusion ability of the compounds and incubation temperature CpA:LA promoted biofilm detachment/removal in all microorganisms	[101]
4	ChCl with amines, alcohols, sugars, and organic acids	*Gram(+): Staphylococcus aureus and Listeria monocytogenes* *Gram-(-) Escherichia coli and Salmonella enteritidis*	Toxicity of DESs containing organic acids: ChCl:A ˃ChCl:Levulinic acid(LeA) ˃ChCl:CA ˃ChCl:MA ˃ChCl: Malic acid (MaA)˃ChCl: tartaric acid (TA) ˃ChCl/*p*-toluenesulfonic acid (PTSA).	[102]
5	BC:AcA BC:McA Polymer BC:AcA and BC:McA	*Gram(+): Staphylococcus aureus* *Gram-(-) Escherichia coli* *Candida albicans*	Toxicity of monomeric DESs higher than polymeric AcA > BC:AcA > BC:McA > BC > McA for *S. Aureus* and *C. Albicans* Similar toxicity for *E. coli*	[104]
6	Menthol:BA Menthol:PhA	*Gram-(-) Escherichia coli* *Gram(+): Staphylococcus aureus* and *Bacillus subtilis*	Similar MIC values for THEDESs.	[105]
7	ChCl with gly, U, EG EAC with gly, U, EG, ZnCl_2_ and MA	*Aspergillus niger* *Cyprinus carpio fish*	MIC values EAC:ZnCl_2_<EAC:MA < ChCl:U <ChCl: EG < EACgly < ChCl:gly Dose-dependent results on Agar diffusion Same trend for fish toxicity	[68]
8	ChCl with gly, EG, DEG, TEG, Frc, Glc, MA, PTSA, ZnCl_2_ and U	*Phanerochaete chrysosporium*, *Aspergillus niger*, *Lentinus tigrinus, Candida cylindracea* *Cyprinus carpio fish*	ChCl with U, gly, Frc, and Glc showed no inhibition to all fungi Highest values of inhibition were found for *C. cylindracea* Only ChCl:ZnCl_2_ was toxic for fish	[109]

The influence of the structure and nature of the HBD component of the DES was studied by applying the Agar diffusion test to twenty different amines, alcohols, sugars, and organic acids in ChCl-based DESs. Tests were performed using two Gram-positive and two Gram-negative bacteria, with results only showing the harmful effect of organic acid-containing DESs (Table 4, entry 4) [102]. This inhibition of bacterial growth was mainly attributed to pH changes whose value decreased with the elongation of the organic acid carbon chain.

Also, the structure of the DESs components has a strong influence on their toxicity. For instance, benzalkonium chloride (BC, a quaternary ammonium antimicrobial agent also known as Zephiran) [103], was combined with acrylic acid (AcA) or methacrylic acid (McA). The antibacterial activity of corresponding DESs and their polymers were assessed. The agar diffusion test using *E. coli*, *S. aureus* and *C. albicans* was chosen as a method to evaluate their toxicity (Table 4, entry 5) [104]. The antimicrobial activity of AcA could be attributed to its acid nature, low molecular weight, and α,β-unsaturation. Reduced inhibition halos were found with McA and their DESs, due to increased structural complexity and decreased linearity that may limit the molecule movement. When this methodology was performed on THEDESs based in menthol combined with three different active pharmaceutical ingredients (APIs), such as benzoic acid (BA) and phenylacetic acid (PhA), the antibacterial capacity was retained compared to each pure API component but was lower to that of pure menthol (Table 4, entry 6) [105].

Besides bacteria, other organisms have been used to determine the DESs toxicities through diffusion methods [106, 107] with the MIC being measured with a broth dilution assay. In general, ChCl-based DESs showed lower toxicity compared to EAC-based DESs, while having MIC values close to those of the HBD components (Table 4, entry 7) [68]. Moreover, the acute toxicity was performed by evaluating the lethal concentration at 50% (LC_50_) of the same DESs on *Cyprinus carpio fish* [108], showing a similar toxic trend. Following this study, the toxicity profile of ten ChCl-based DESs towards four fungi strains and *Cyprinus carpio fish* was conducted (Table 4, entry 8) [109]. Only ChCl:ZnCl_2_, ChCl:MA and ChCl:PTSA exhibited antifungal inhibition toward all fungi strains. In general, most tested DESs displayed similar or lower inhibition zones on the selected fungi than their components.

Also, viruses (Herpes Simplex Virus Type-1 and Type-2 (HSV-1 and HSV-2), have also been included in DESs toxicity studies. A ChCl and geranate (CAGE) DES was synthesized through salt metathesis of 1:2 M ratio choline bicarbonate and geranic acid [110]. The antimicrobial and antifungal activity of CAGE was explored against 38 bacteria and seven fungi, together with the two previously mentioned viruses, using standard Clinical and Laboratory Standards Institute (CLSI) broth microdilution methodology [111]. The main results from this study confirmed that CAGE possessed a broad-spectrum antimicrobial activity, especially against *Propionibacterium acnes.* Moreover, even with diluted DES, fungi and viruses were neutralized.

Undoubtedly, the most complete studies about DESs toxicity are those in which microbial incubation is carried out in a culture medium (preferably, liquid media), to later observe the growth. Following this methodology, a complete study was conducted with *E. coli* in LB culture medium, in the presence of AcChCl:A at concentrations up to 600 nm [112]. By using this methodology, the pH of the medium is continuously monitored, permitting an assessment of whether the toxicity is due to the DES's nature or variations in pH during the assay. Recent studies suggest that optimizing the buffering of the culture media or using preadapted strains or extremophilic microorganisms instead of standard microbial strains main may allow the pH changes seen in those toxicity assays to be overcome.^112,113.^

In silico models provide an excellent means of assessing the toxicity of DESs in a global approach. One of the models used in this context consists of the study of quantitative structure-toxicity relationships (mtk-QSTR). Thus, mtk-QSTR modelling of 572 different DESs was carried out under experimental conditions, with high precision (around 90%). Besides highlighting the properties of DESs that influenced their potential toxicity, this model allowed classifying HBDs based on their toxic contributions (DESs-organic or inorganic acids > DESs-amides or sugars> (DESs-alcohols) [113].

## 5 Conclusions

From the analysis here displayed, the following main conclusions can be highlighted: i) a significant number of DESs are less environmentally friendly than initially thought probably due to a synergistic effect of their components, consequently their potential uses at a large scale should be revised in order to avoid environmental pollution; ii) toxicity/biodegradability tests based on antibiogram in solid media are not accurate to quantify DESs toxicity due to their low diffusion as a consequence of their high viscosity; iii) more studies about toxicity/biodegradability should be conducted in the near future using a wider spectrum of different types of microbial/cellular phenotypes for every single DESs tested (Gram-positive *vs*. Gram-negative; prokaryote *vs.* eukaryote and among prokaryotes Archaea and Bacteria must be considered; animal cell *vs*. plant cell, etc.). Only in this way it can be categorically stated whether a particular DES is toxic, even in cases in which such DESs could enter the food chain.

## Declarations

### Author contribution statement

All authors listed have significantly contributed to the development and the writing of this article.

### Funding statement

Dr. Rosa María Martínez- Espinosa was supported by Conselleria de Innovación, Universidades, Ciencia y Sociedad Digital, Generalitat Valenciana [PROMETEO/2021/055].

Gabriela Guillena Townley was supported by University of Alicante [VIGROB-173], MICINN Spain [PGC2018-096616-B-I00].

This work was supported by University of Alicante [VIGROB-309], MICINN Spain [RTI2018-099860-B-I00].

### Data availability statement

Data included in article/supp. material/referenced in article.

### Declaration of interest's statement

The authors declare no competing interests.

### Additional information

No additional information is available for this paper.
